# Exploring the Pharmacological Potential of Metformin for Neurodegenerative Diseases

**DOI:** 10.3389/fnagi.2022.838173

**Published:** 2022-04-26

**Authors:** Ming-Rui Du, Quan-You Gao, Chen-Lin Liu, Lin-Ya Bai, Tian Li, Fei-Long Wei

**Affiliations:** ^1^Department of Orthopedics, Tangdu Hospital, Fourth Military Medical University, Xi’an, China; ^2^State Key Laboratory of Cancer Biology, Biotechnology Center, School of Pharmacy, Fourth Military Medical University, Xi’an, China; ^3^School of Basic Medicine, Fourth Military Medical University, Xi’an, China

**Keywords:** metformin, neurodegeneration, AMP-activated protein kinase, narrative review, pharmacology

## Abstract

Metformin, one of the first-line of hypoglycemic drugs, has cardioprotective, anti-inflammatory and anticancer activities, in addition to its proven hypoglycemic effects. Furthermore, the preventive and therapeutic potential of metformin for neurodegenerative diseases has become a topic of concern. Increasing research suggests that metformin can prevent the progression of neurodegenerative diseases. In recent years, many studies have investigated the neuroprotective effect of metformin in the treatment of neurodegenerative diseases. It has been revealed that metformin can play a neuroprotective role by regulating energy metabolism, oxidative stress, inflammatory response and protein deposition of cells, and avoiding neuronal dysfunction and neuronal death. On the contrary, some have hypothesized that metformin has a two-sided effect which may accelerate the progression of neurodegenerative diseases. In this review, the results of animal experiments and clinical studies are reviewed to discuss the application prospects of metformin in neurodegenerative diseases.

## Introduction

Metformin (dimethyl biguanide hydrochloride) is a natural derivative of guanidine, which is found in French cloves (Galega officinalis). It was originally described as a hypoglycemic and anti-malarial drug. The results of a multi-center randomized controlled trial (RCT) in the United Kingdom have profoundly influenced metformin as a treatment for type 2 diabetes (T2D) (No authors listed, 1998). Clinical studies have shown that enhanced glycemic control with metformin reduces the incidence of diabetes-related endpoints and all causes of mortality. In addition, metformin has been shown to extend life span in different animal models (Barini et al., 2016).

Metformin has been shown to decrease intestinal glucose absorption (Pryor and Cabreiro, 2015) and fasting plasma insulin level, increase insulin sensitivity, promotes muscle glucose uptake, activates AMP-activated protein kinase, and reduces liver gluconeogenesis. These helps lower blood glucose concentration without causing obvious hypoglycemia (Inzucchi et al., 2015). Also, metformin can lower the risk of cardiovascular disease (Luo et al., 2019), improve endothelial function in female patients with polycystic ovary syndrome(PCOS) (Heidari et al., 2019), and may prevent or cure age-related diseases (Orchard et al., 2005). More recently, other researches have suggested that metformin could inhibit many cancers, including lung, stomach, and endometrial cancers (Rizos and Elisaf, 2013).

Neurodegenerative diseases are caused by necrosis or loss of function of neurons, including Alzheimer’s disease (AD), Parkinson’s disease (PD) and Huntington’s disease (HD). When neurodegenerative disease occurs, the number of neurons in the brain decreases and synaptic signaling is disrupted. Aging cells become unable to maintain proteins in the correct folding state. This leads to decreased protein homeostasis in the brain, imbalance of epigenetic modifications such as DNA methylation, abnormal signal transduction between cells, inflammatory response and dysfunction of insulin in the brain. All these further aggravate the development of neurodegenerative diseases (Campbell et al., 2018). Increasing evidence shows that metformin can trigger autophagy, prevent oxidation, reduce neuroinflammation, and play a role in neuroprotection and neurorepair for a variety of nervous system diseases. In order to explore the prospect of metformin in neurodegenerative diseases, this paper reviewed the molecular mechanism of metformin, the progress of animal experimental research and some clinical retrospective studies.

## Mechanisms and Signaling Pathways of Metformin

### AMPK Signaling

**FIGURE 1:**
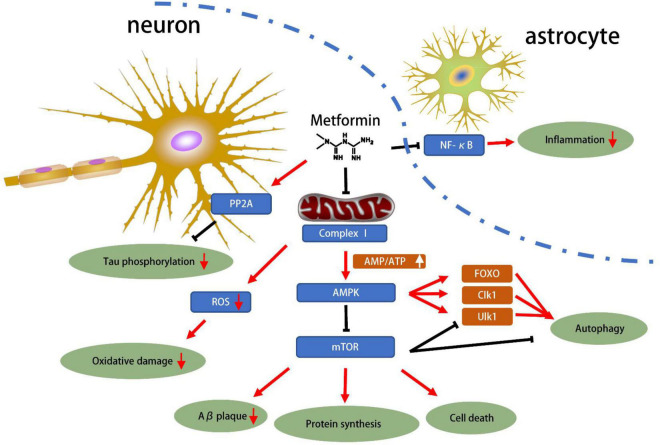
Mechanisms of metformin related to neuroprotection for NDD. The progression of neurodegenerative diseases has been shown to be associated with Tau phosphorylation, oxidative stress, amyloid protein accumulation and inflammation. Metformin can inhibit the NF-κB pathway, inhibit the astrocyte inflammation. In neurons, metformin directly inhibits Tau phosphorylation through PP2A. Most importantly, metformin inhibits respiratory chain complex 1, which inhibits energy production. This reduces ROS production, increases the AMP/ATP ratio, and then activates AMPK, which in turn inhibits Aβ plaque, protein synthesis, cell death and autophagy pathways. In general, metformin has great potential for neuroprotection based on current preclinical studies.

The protective effect of metformin had been summarized in Figure 1. AMPK plays an important role of cellular energy metabolism in T2D patients. Activated AMPK regulates glucose and lipid metabolism, autophagy, apoptosis and other cellular processes (Dengler, 2020). Metformin acts on the respiratory chain complex I in the mitochondria, inhibiting ATP production, thereby increasing cytoplasmic ADP/ATP and AMP/ATP ratios. When the balance is disrupted and the ratio is increased, AMPK promotes catabolic pathways, increases ATP production and inhibits ATP consumption to restore cellular homeostasis (Rena et al., 2017). Besides, metformin might activate AMPK through a lysosomal pathway (Jia et al., 2020). Activated AMPK inhibits fat synthesis and promotes fat utilization through phosphorylation of ACC1 and ACC2, thereby reducing hepatic lipid storage and improving hepatic insulin sensitivity (Rena et al., 2017).

### Insulin Signaling

Insulin in the brain can regulate nutrient dynamic balance and cognitive function and has neurotrophic, neuroregulatory and neuroprotective effects. Insulin achieves its function by activating insulin receptor (IR) and its downstream pathway. IR is a family of tyrosine kinase receptors. The binding of islet to IR can phosphorylate IR dimer tyrosine, which in turn phosphorylates insulin receptor substrate (IRS), activating downstream phosphatidylinositol 3-kinase, PI3K/protein kinase B and mitogen-activated protein kinase (MAPK) to produce downstream effects (Manning and Cantley, 2007; Manning and Toker, 2017). Insulin resistance is associated with disturbances of upstream and downstream Akt signaling. During the progression of neurodegenerative changes, insulin gene expression level and IR or IRS protein expression are significantly decreased (Rivera et al., 2005). These results suggest that insulin resistance may play a role in the development of neurodegenerative diseases. Metformin is a commonly used insulin sensitizer in clinic, which can improve the sensitivity of IR to insulin. Metformin inhibits the PI3K/Akt signaling pathway through AMPK (Zakikhani et al., 2010). In addition, metformin down-regulates the expression of insulin and IGF-1 receptors and reduces the phosphorylation of insulin receptors, including IRS-1 (Sarfstein et al., 2013). Epidemiological studies have observed that metformin treatment reduces the risk of dementia in patients with diabetes (Zhou et al., 2015).

### Antioxidant Mechanisms of Metformin

Reactive oxygen species (ROS) are by-products of aerobic cell energy metabolism and catecholamine neurotransmitter metabolism, which need to be eliminated in neuron. An imbalance in the production and clearance of ROS may lead to oxidative stress in biological systems, which may contribute to the development of neurodegenerative diseases. As a result, the antioxidant system is crucial to prevent any influence caused by ROS overproduction (Rotermund et al., 2018). Metformin plays a role in these pathways involved in disease progression by inhibiting complex I in mitochondria to slow oxidative phosphorylation and inhibit gluconeogenesis. This helps reduce the oxidative burden on neurons by minimizing Nicotinamide adenine dinucleotide (NADH) utilization (Rotermund et al., 2018). An et al. (2016) found that metformin had antioxidant effects on human umbilical vein endothelial cells (HUVECs) *via* an AMPK-dependent pathway. Metformin promoted endothelial nitric oxide synthase (eNOS) re-coupling *via* upregulation of GTPCH1 (guanosine 5′-triphosphate cyclohydrolase 1) and BH4 (tetrahydrobiopterin) levels. In addition, the level of nicotinamide adenine dinucleotide phosphate (NADPH) oxidase subunit p47-phox was down-regulated, thereby reducing ROS generation and BH4 oxidation. Metformin can also act as an antioxidant through other pathways. Metformin can activate fork head box O 3 (FOXO3), reducing ROS/RNS level in immune cells (Hartwig et al., 2021). Importantly, metformin has a concentration-dose effect, and high concentrations of metformin can lead to cell apoptosis through the accumulation of ROS. This metformin-induced effect may depend on its inhibition of nuclear factor erythroid 2 like 1 (NFE2L1) which crucially regulates cellular redox homeostasis (Gou et al., 2021). Cisplatin combined with metformin significantly down-regulated Nrf2 by inhibiting the Ras/Raf/ERK pathway in lung cancer cells, which promoting ROS-mediated apoptosis in the cells (Huang et al., 2020).

### Anti-inflammatory Mechanisms of Metformin

Neuroinflammation widely exists in various central nervous system diseases and is closely related to cognitive dysfunction. Studies have shown that neuroinflammation is involved in the pathogenesis of neurodegenerative diseases. Abnormal activation of microglia cells can lead to immune dysfunction and inflammation, in which microglia cells release inflammatory mediators and damage normal surrounding nerve tissue, gradually leading to degeneration and death of neurons (Zajda et al., 2020). Due to the continuous abnormal immunity of microglia, chronic neuroinflammation leads to the destruction of neurons and synaptic structures, resulting in the development of neurodegenerative diseases such as AD, PD, and HD.

Researches have claimed that metformin inhibits NF-κB (nuclear factor kappa-light-chain-enhancer of activated B cells) signaling and the production of pro-inflammatory cytokines of different cell types (Zhou et al., 2021). Docrat et al. (2021) demonstrated that NF plays a neuroprotective role by regulating protein phosphatase 2A (PP2A) dephosphorylation of Tau protein mediated by miR-141. In addition, metformin can be anti-inflammatory by restraining NF-κB mediated NLRP3 inflammase-associated transcription. Metformin may activate the PP2A pathway by decreasing the phosphorylation of PP2Ac at Y307 and disrupting the PP2AC-A4-MID1 interaction. In this way, inflammatory response of primary cardiomyocytes induced by high glucose stimulation were inhibited (Cheng and Li, 2020). Metformin has the ability to activate the AMPK signaling pathway while inhibiting the inflammatory response of bovine mammary epithelial cells. Meanwhile, metformin inhibited the acetylated histone H3 to inactivate the NF-κB, which in turn inhibits the expression of pro-inflammatory genes (Xu T. et al., 2021). Metformin inhibits the interaction between Caveolin1 and AMPKA, thereby reducing hyperglycemic stress and inflammation (Wang et al., 2021). In addition, metformin can reduce inflammatory response by inhibiting translocation of NF-κB, phosphorylation of ERK1/2 and p38 MAPK and reducing the production of inflammatory cytokines (TNFα, IL1β and IL6) (Tao et al., 2018).

### Autophagy-Inducing Mechanisms of Metformin

Autophagy is an important cellular function that mediates the degradation of dysfunctional proteins and organelles in cells. In neurodegenerative diseases, autophagy is often dysregulated, promoting abnormal protein accumulation and organelle dysfunction (Moujalled et al., 2021). Researches have shown that metformin can promote autophagy *in vivo* and *in vitro*, to reduce Tau hyperphosphorylation and improve cognition, through APMK-dependent pathways (Chen et al., 2019). In addition, metformin can inhibit the infection activity of prions by activating autophagy pathways (Abdelaziz et al., 2020).

The traditional pathways of autophagy include PRKA (protein kinase activation)-mTOR-ULK1 signaling and the NADC-the sirtuin histone/protein deacetylase inhibitor (SIRT1) FOXO pathway. Interestingly, metformin has been confirmed to activate both PRKA and SIRT1 (Song et al., 2015). In addition, Bharath et al. (2020) discovered that metformin reduced age-related inflammation by promoting autophagy and normalizing mitochondrial function. It is worth mentioning that the activation of autophagy by metformin is not unidirectional. In tumor cells, metformin can exert anti-tumor effects through other interesting pathways of inhibition of autophagy. The GRP78 is a key driver of bortezomib-induced autophagy. However, studies have shown that metformin can inhibit glucose-regulated protein 78 (GRP78), which breaks the cell defense state and enhances the efficacy of bortezomib-induced autophagy against myeloma (Jagannathan et al., 2015).

### Anti-aging Mechanisms of Metformin

Studies have shown that metformin has beneficial effects on metabolic and cellular processes closely related to the development of age-related diseases, such as regulating glucose metabolism, improving insulin sensitivity, and inhibiting oxidative stress, suggesting that metformin may have anti-aging potential (Barzilai et al., 2016). Metformin has been shown to regulate receptors for cytokines, insulin, IGF-1, and adiponectin, all of which are activated with age and associated with longevity. Metformin inhibits intracellular inflammatory pathways, activates AMPK, and increases inhibition of mTOR, which may be a major target for regulating aging. Metformin inhibits mitochondrial complex 1, regulates oxidative stress and clears senescent cells. Together, these processes affect inflammation, cell survival, stress defense, autophagy, and protein synthesis, which are major biological outcomes associated with aging/longevity (Barzilai et al., 2012). Although metformin has been shown to extend life, this may be because it reduces mortality from diseases, not just anti-aging. More studies are needed to clarify metformin’s anti-aging potential.

## Metformin and Neurodegenerative Diseases

Metformin is a drug with an extensive range of targets, whose neuroprotective effects have been widely reported. Positive results have been achieved in animal models, as shown in Table 1. Metformin not only influences the occurrence and development of neurodegenerative diseases by reducing blood glucose, but it also has promising anti-oxidative, anti-inflammation, anti- senescence, inhibition of Tau protein phosphorylation, and other effects. Even though there have been many researches revealing the neuroprotective function of metformin, it is worth noting that some experimental results have been contradictory. The safety, multi-target, and mechanism of metformin’s effect on neuronal longevity have not been fully elucidated. Different experimental models, different combinations of drugs, and different genetic backgrounds of the experimental population could all contribute to this. We will conduct a systematic review of metformin’s potential protective role in neurodegenerative diseases in part.

**TABLE 1:** Effects of metformin on neurodegenerative disease models.

Study	Disease	Species	Models’ features	Neuroprotective effect	Mechanisms of action
Pilipenko et al., 2020	AD	Rat	STZ	Improveing microglia proliferation and astroglia proliferation, retaining hippocampal synaptic plasticity, inhibiting acetylcholinesterase activity.	Improveing glucose transport, uptake and metabolism in the brain.
Chen et al., 2019; Greco et al., 2009	AD	Mouse	db/db mice (BKS.Cg-Dock7m + / + Lepr^db^/J)	Reducing hyperphosphorylated tau protein, alleviating muscular dystrophy and improving cognitive impairment.	Enhancing autophagy activity in an AMPK dependent manner
Chen et al., 2021	AD	Mouse	Injection of brain extracts containing tau.	Reducing Aβ deposition and tau lesions.	Enhancing microglia autophagy activity
Saffari et al., 2020	AD	Rat	STZ	Restore learning and memory function and reducing cytokines in hippocampus	Anti- neuroinflammation
Lu et al., 2020	AD	Mouse	APP^swe^/PS1^dE9^(APP/PS1) transgenic mice.	Improving learning and memory ability and neurological dysfunction.	Promoting the protein expression of P-AMPK and IDE, reducing the expression of Aβ, inhibiting oxidative stress and neuroinflammation.
El-Ghaiesh et al., 2020	PD	Mouse	Rotenone.	Improving motor function, promoting the distribution and expression of glutathione, Nrf2, hemogloxygenase-1 and thioredoxin in the striatum, and increasing the number of tyrosine hydroxylase positive neurons.	Antioxidant by activating of AMPK-FOXO3 signal and inhibiting of VEGF.
Tayara et al., 2018	PD	Rat	LPS.	Inhibiting the activation of microglia.	Antioxidant by decreasing the phosphorylated forms of MAPKs and ROS generation through the inhibition of the NADPH oxidase enzyme.
Saewanee et al., 2021	PD	Elegans	6–OHDA	Restoring food perception, inhibiting α-synuclein aggregation, and up-regulating dopamine synthesis and antioxidant genes.	Antioxidant.
Katila et al., 2021	PD	Mouse	MPTP.	Decreasing reactive oxygen species level and restoring mitochondrial function.	Antioxidant by AKT-Nrf2 mediated transcriptional upregulation.
Gomez-Escribano et al., 2020; El-Ghaiesh et al., 2020	HD	Elegans	C.elegans models of polyQ toxicity.	Metformin and salicylate synergistically reduce polyQ aggregation.	AMPK and autophagy pathways
Sanchis et al., 2019	HD	Mouse and worm	zQ175 mouse model and worm models of polyglutamine toxicity.	Reducing motor and neuropsychiatric phenotypes, reducing nuclear aggregation of mHtt in the striatum, reducing glial activation, and restoring neurotrophic factors.	Reduction of polyglutamine accumulation through AMPK and lysosomal pathways.
Vazquez-Manrique et al., 2016	HD	Elegans and mouse	C. elegans model of neuronal dysfunction (Tourette et al., 2014) and mouse striatal cells expressing full-length mutant Htt.	Activation of metformin and AMPK protects against neurological dysfunction caused by mHtt.	AMPK signaling.

*MPTP, 1-Methyl-4- phenyl-,2,3,6tetrahydropyridine; Met, Metformin; STZ, streptozocin; 6–OHDA, 6-hydroxydopamine; LPS, lipopolysaccharide; mHtt, mutant Huntington protein.*

### Alzheimer’s Disease and Metformin

Alzheimer’s disease is a progressive neurodegenerative disease among older individuals. The main histopathological feature is the extracellular deposition of amyloid B(Aβ) with microglia and astrocytes surrounding, leading to further neurotoxicity and organelle damage. This eventually leads to mitochondrial dysfunction, neuroinflammation and other pathological events (Yang and Zhang, 2020).

Studies have shown that dysregulation of AMPK is associated with AD. AMPK regulates Aβ production and tau phosphorylation, which is considered to be the molecular pathology of AD. However, there is paradox about the effect of AMPK on AD. On the one hand, in cultured neurons, AMPK activation inhibits Aβ production and tau phosphorylation, and reduces extracellular Aβ accumulation (Greco et al., 2009; Vingtdeux et al., 2010). On the other hand, AMPK-related signaling is activated in APP/PS1 transgenic AD mouse neurons, and AMPK inhibitors reduces ALR-related protein expression levels, thereby ameliorating memory deficits (Du et al., 2019). Oliveira et al. found that metformin activation of AMPK can reduce neuroinflammation and neuronal damage, improve cognition, and improve the symptoms of AD (Oliveira et al., 2021). But the underlying molecular/cellular mechanisms needed further clarification.

Insulin resistance is also thought to be a factor in the development of AD. Insulin resistance leads to dephosphorylation and activation of GSK-3β, resulting in increased production of Aβ, which in turn increases tau phosphorylation associated with NFT formation, contributing to many of the pathological features associated with AD. In addition, metformin is found to reduce Aβ level in APP/PS1 mice through insulin-degrading enzyme (IDE) pathway (Wang et al., 2020). Zhang et al. (2021) found that metformin can improve the AD-like phenotype induced by MC-LR by activating MTOR dependent PP2A and GSK-3β, thereby inhibiting tau phosphorylation at Ser202.

Studies have proved the close relationship between T2D and AD, which are believed to share some common pathophysiological characteristics (Zhou et al., 2015). Metformin therapy has been observed to reduce the risk of dementia in diabetes (Saffari et al., 2020). Elsewhere, metformin can improve the learning and memory capacity and neurological dysfunction of APP/PS1 mice by reducing Aβ (Lu et al., 2020). Farr et al. (2019) obtained the same conclusion that metformin facilitated learning and memory capacity in SAMP8 mice by reducing APPC99 and pTau proteins. High glucose can induce brain astrocyte inflammation and endoplasmic reticulum stress, which can promote the progression of AD. However, inflammation and endoplasmic reticulum stress are inhibited in metformin-pretreated astrocytes. This process may be mediated by metformin through inhibition of the formation of Caveolin1/AMPKα complex (Wang et al., 2021).

Metformin can improve microglia autophagy in APP/PS1 mice. Tau aggregates are injected into APP/PS1 mouse brain to promote the pathological progression of AD. Interestingly, metformin mitigates this pathological process by improving microglial autophagy, increasing the number of microglias around Aβ plaques, and promoting NP Tau phagocytosis (Chen et al., 2021). Xu X. et al. (2021) Found that metformin induces lysosomal dependent autophagy through AK1-IKK α/βHsc70 signaling, reversing the molecular and behavioral phenotypes of AD.

The other causes of AD are abnormal cholinergic systems and hippocampal synaptic transmission. Early AD is usually manifested by abnormal cholinergic function. The apoptosis of cholinergic neurons leads to a decrease in acetylcholine secretion. The deficiency of acetylcholine leads to cortical and hippocampal dysfunction, resulting in abnormal memory and movement of the body. Metformin can improve synaptic defects, memory problems and enhance cognition in animal models of AD (Pilipenko et al., 2020; Saffari et al., 2020). In the STZ-induced rat model, metformin can preserve the synaptic plasticity of the hippocampus, normalize the acetylcholine cleavage in the cortex and hippocampus tissues, and inhibit acetylcholinesterase activity (Pilipenko et al., 2020). This suggests that metformin can prevent neurogenic diseases potentially. In addition, Chen et al. (2020) revealed that metformin treatment can increase glutamate transmission rather than GABA transmission, which may be beneficial for the patients of AD or other relative brain disorders. Cyclin-dependent kinase 5(CDK5) is a serine/threonine kinase regulated by P35/P39 that plays an important role in synaptic plasticity and cognitive behavior. However, abnormal activation of CDK5 can lead to synaptic inhibition, resulting in the early pathological changes of Alzheimer’s disease (AD). A et al. found that metformin prevents Cdk5 overactivation by inhibiting the calpain-dependent cleavage of p35 into p25, thus preventing the onset of AD (Wang et al., 2020).

In the study involving individuals aged over 50 years in a Taiwan database, Hsu et al. (2011) suggested that metformin reduced the risk of dementia by 24% in T2D patients. When combined with sulfonylureas, it reduced that risk by 35%. Aaron et al. investigated its influence on neuroimaging and cognitive ability. Cerebral blood flow wasn’t changed significantly after metformin treatment, but there was significant improvement in executive function and a tendency to improve learning/memory and attention (Koenig et al., 2017). Clinical data suggest that metformin has a dual effect on cognition, which may be caused by B12 deficiency (Kuan et al., 2017). A prospective trial conducted in Australia (Moore et al., 2013) found that metformin is related to cognitive dysfunction, which can be alleviated by vitamin B12 and calcium. Campbell et al. (2018) advised that metformin can be used as the first-line therapy for diabetics to prevent dementia or AD. However, the successive use of metformin reduces vitamin B12 levels, which may account for the failure of metformin’s protective effect. Therefore, it is recommended that elderly patients with diabetes should be supplemented with vitamin B12 when taking metformin. Overall, more definitive experimental data are needed to determine whether metformin protects against dementia in non-diabetic patients.

### Parkinson’s Disease and Metformin

Parkinson’s disease is a neurodegenerative disease mediated by a variety of factors. The main pathological features are the advanced decrease of dopaminergic neurons in the brain nigra and their projection to neurons in the striatum. Metformin has been shown to alleviate PD progression in a variety of ways.

The two cardinal pathologies of PD are degeneration of SNc-DA neurons and the accumulation of αSyn aggregates in Lewy bodies. AMPK subunits α-1 and α-2 have neuroprotective effects against αSyn toxicity, almost completely preventing the loss of dopaminergic neurons. Metformin affects neuronal function in rodent PD models through AMPK-mediated phosphorylation of αSyn (Perez-Revuelta et al., 2014). However, AMPK seems to be a double-edged sword in PD progress. Overactivated AMPK promotes the accumulation of α-SYN (184), resulting in SYN binding to GTPase pike-L and dopaminergic cell death (Ng et al., 2012). Sardoiwala et al. (2020) constructed polynanocarrier for Metformin delivery (Met Encapsulated PDANPs) to facilitate metformin to cross the blood-brain barrier. Then they found that metformin Encapsulated PDANPs promotes EZH2-mediated ubiquitination of α-SYN at PSer129, triggering the ubiquitin-proteasome degradation pathway of α-SYN, enhancing the neuroprotective effect of metformin and providing a new idea for optimization of metformin (Sardoiwala et al., 2020).

The BCAT-1 gene knockout Caenorhabditis elegans showed PD-like symptoms such as neurodegeneration with age, advanced dyskinesia and increased mitochondrial respiration (Mor et al., 2020; Sohrabi et al., 2021). Metformin can reduce mitochondrial respiration of BCAT-1 knockout Caenorhabditis elegans to a controlled level while improving motor function and neuronal activity (Mor et al., 2020). This suggests that metformin may offer a new treatment strategy against PD by inhibiting mitochondrial respiration. Metformin has neuroprotective effects on 6-hydroxydopamine-induced dopaminergic neurodegeneration and β -synuclein aggregation in nematode models of Parkinson’s disease. However, metformin do not significantly increase the mean or maximum lifespan of model animals, and the exact mechanism remains to be further studied (Saewanee et al., 2021).

Rotenone can cause oxidative stress and mitochondrial dysfunction, which are the main pathologic features of Parkinson’s disease. Therefore, it is often used to induce experimental Parkinson’s disease in cells and animals. Katila et al. (2021) found that metformin induces antioxidant system through NRF2-mediated transcriptional up egestion, thereby inhibiting intracellular oxidative stress, thereby restoring mitochondrial dysfunction and energy loss, and avoiding rotenone-induced SH-SY5Y cell death. In another rotenone-induced mouse model of PD, MTF reduces VEGF and Caspase3 activity by activating the AMPK-FOXO3 pathway, improving antioxidant capacity in the striatum and improving motor function in mice. In addition, MTF increases the number of tyrosine hydroxylase (TH) staining neurons in substantia nigra and striatum neurons, delaying the progression of PD (El-Ghaiesh et al., 2020).

Some studies have shown that neurotrophic factors such as glial cell-derived neurotrophic factor (GDNF) and brain-derived neurotrophic factor (BDNF) upregulate tyrosine hydroxylase (TH), improve movement disorders and provide neuroprotection in animal models of Parkinson’s disease (Gash et al., 2005; Lindholm and Saarma, 2021). Katila et al. (2020) indicated that by promoting the phosphorylation of tyrosine hydroxylase (TH), metformin simultaneously increases brain-derived and glial cell-derived neurotrophic factor (GDNF), thereby activating the cell survival signaling pathways.

Tayara et al. (2018) proposed that using metformin in diabetic populations at risk of PD be adequately evaluated. Their findings suggest that metformin has a paradoxical effect on nerves, with both beneficial and harmful effects. The authors further demonstrated the its anti-inflammatory function and overall immunosuppressive influence on microglia. However, Tayara et al. (2018) suggested that metformin was harmful to the dopamine system (Wahlqvist et al., 2012). According to the study, patients of T2D doubled the risk of PD, and with anti-hyperglycemia treatment, the risk of PD decreased to 1.3-fold. When sulfonylureas were used in combination with metformin, the HR decreased to 0.4, suggesting that sulfonylureas, in combination with metformin, had a protective effect on PD. A 12-year retrospective cohort study demonstrated that metformin increased risk of dementia and PD. Therefore, the authors suggest that successive metformin treatment in type 2 diabetes may contribute to the progress of neurodegenerative diseases (Kuan et al., 2017). However, the authors did not take into account vitamin B12 levels in the population, which may have influenced their final conclusions.

### Huntington’s Disease and Metformin

Huntington’s disease (HD) is a dominant inherited neurodegenerative disorder in which patients express high levels of Htt due to abnormal amplification of the first exon CAG of the gene encoding Huntington protein. When the CAG triplet of Htt is repeated more than 35 times, the mutant Huntingtin(mHtt) containing an abnormally long polyQ chain is produced. Improper folding and aggregation of mHtt produce toxic effects on striatum neurons, resulting in impaired motor coordination, chorea and progressive deterioration of cognitive function (Walker, 2007). There is no resultful therapy, but studies have shown that metformin has great therapeutic potential for this disease. A global observational clinical study of HD has assessed the effects of metformin on cognition, with linear analysis finding that metformin was related to the better cognitive ability of patients with HD (Hervas et al., 2017).

Animal experiments have shown that metformin can prolong the survival time of HD transgenic male mice (Ma et al., 2007). Dysfunction of the visual cortical circuits is one manifestation of the early effects of HD. Arnoux et al. (2018) found that in presymptomatic HD mice, neurons in the visual cortex were hyperactive and increased simultaneously, accompanied by behavioral abnormalities. Interestingly, metformin reduced mHtt *via* the MID1/PP2A complex and reduced S6 phosphorylation, which completely restored the mice’s early network activity pattern and behavioral abnormalities. These results show the perspective on the early prevention and treatment of HD with metformin.

The regulation of energy homeostasis by adenosine monophosphate activated kinase (AMPK) is crucial in cell survival and biological longevity, and its activation was proved *in vivo* and *in vitro* to be beneficial to the recovery of HD. Vazquez-Manrique et al. (2016) confirmed that the AMPK pathway is over activated in Caenorhabditis elegans and mouse HD models, and they suggested that metformin may work as a protective role in the early progression of HD. This conclusion was confirmed by Jin et al. (2016). They found that metformin may weaken the toxic effect of Htt through activating the AMPK pathway. In addition, Gomez-Escribano et al. (2020) found that the low doses of metformin and salicylate could synergistically activate AMPK and alleviate the neurotoxic effects of extended polyQ and α-synuclein, which provided a new idea for the combination of metformin.

Sanchis et al. (2019) observed that metformin alleviated HD symptoms in animal models in many ways. In the zQ175 Mouse model of HD, they found that metformin reduced the nuclear aggregation of mHtt in the striatum, partial recovery of neurotrophic factor expression, and decreased glial cell activation. In addition, metformin reduced polyglutamine aggregation and neuronal damage in a nematode model by an AMPK and lysosome dependent mechanism (Sanchis et al., 2019).

### Other Neurodegeneration Diseases and Metformin

Sporadic cerebral amyloidosis (CAA) is caused by the deposition of amyloid (Aβ) protein in the blood vessels of the brain. It is the main cause of recurrent cerebral hemorrhage and dementia in the elderly (Viswanathan and Greenberg, 2011). Inoue et al. gave CAA and T2 DM mixed mouse model (APP23-ob/ob) metformin treatment, that Aβ in the cerebral cortex and hippocampus of APP23ob/ob mice significantly decreased at the 15th month. But IDE (Insulin-degrading enzyme) were significantly increased in the hippocampus, which is a kind of a Aβ-degrading enzyme. These results suggest that metformin can promote degradation of Aβ and reduce AB deposition in the cerebrovascular system (Inoue et al., 2021).

NPC1(Niemann–Pick disease type C1) is a neurodegenerative inherited recessive disorder caused by mutations in the NPC1 or NPC2 genes, characterized by disturbed intracellular transport of cholesterol and other lipids, resulting in the accumulation of unesterified cholesterol in the central nervous system (Vanier, 2015). HPβCD (2-Hydroxypropyl-β-cyclodextrin) is being developed to treat NPC1. Du et al. (2021) found that combination therapy of HPβCD and metformin in NPC1−/− mice reduced inflammatory responses in NPC1−/− mice and inhibited the release of pro-inflammatory cytokines in the organs. Notably, although the combination therapy did not prolong the lifespan of the mice and metformin did not reduce free cholesterol levels in NPC1 –/– brain tissue or fibroblasts, it provided a valuable exploration for the treatment of NPC1.

## Conclusion

Nowadays, preventive and therapeutic function of metformin on neurodegenerative diseases have been extensively studied. Metformin can promote autophagy, prevent mitochondrial dysfunction, reduce neuroinflammation, and play a role in neuroprotection and neurorepair. However, some scholars believe that the efficacy and safety of metformin applying to neurodegenerative diseases needs further investigation. This may be due to differences in animal models and different drug combination effects. The clinical research progress of metformin in the therapy of neurodegenerative diseases is slow, most of which are the prospective or retrospective clinical studies. Large multicenter randomized controlled trials are still lacking. Metformin has two roles in the treatment of neurodegenerative diseases, according to current clinical research findings. This might be because Metformin can cause vitamin B12 deficiency among T2D patients which presents with diverse clinical presentations like cognitive impairment and dementia. Therefore, the neuroprotective ability of metformin may be enhanced by higher levels of vitamin B12. This review supports that metformin can potentially serve for the prevention and therapy of neurodegenerative diseases, which has been demonstrated by the existing literature on mechanism studies and animal experiments. However, good animal models and large sample randomized controlled trials are urgently needed to provide more adequate clinical evidence support.

## Author Contributions

M-RD and Q-YG: writing the original version. C-LL and L-YB: critical revision and corrections. F-LW and TL: idea, revision, and supervision. All authors contributed to the article and approved the submitted version.

## Conflict of Interest

The authors declare that the research was conducted in the absence of any commercial or financial relationships that could be construed as a potential conflict of interest.

## Publisher’s Note

All claims expressed in this article are solely those of the authors and do not necessarily represent those of their affiliated organizations, or those of the publisher, the editors and the reviewers. Any product that may be evaluated in this article, or claim that may be made by its manufacturer, is not guaranteed or endorsed by the publisher.
